# Embryonic Stem Cell Markers

**DOI:** 10.3390/molecules17066196

**Published:** 2012-05-25

**Authors:** Wenxiu Zhao, Xiang Ji, Fangfang Zhang, Liang Li, Lan Ma

**Affiliations:** 1 Life Science Division, Graduate School at Shenzhen, Tsinghua University, Shenzhen 518055, China; Email: winniezhao@sz.tsinghua.edu.cn (W.Z.); hijiri_byakuren@126.com (X.J.); fng.zhang1@gmail.com (F.Z.); liliang819@gmail.com (L.L.); 2 Department of Biological Sciences and Biotechnology, Tsinghua University, Beijing 100084, China

**Keywords:** embryonic stem cell, marker, tumor stem cell, surface markers, lectin, peptides

## Abstract

Embryonic stem cell (ESC) markers are molecules specifically expressed in ES cells. Understanding of the functions of these markers is critical for characterization and elucidation for the mechanism of ESC pluripotent maintenance and self-renewal, therefore helping to accelerate the clinical application of ES cells. Unfortunately, different cell types can share single or sometimes multiple markers; thus the main obstacle in the clinical application of ESC is to purify ES cells from other types of cells, especially tumor cells. Currently, the marker-based flow cytometry (FCM) technique and magnetic cell sorting (MACS) are the most effective cell isolating methods, and a detailed maker list will help to initially identify, as well as isolate ESCs using these methods. In the current review, we discuss a wide range of cell surface and generic molecular markers that are indicative of the undifferentiated ESCs. Other types of molecules, such as lectins and peptides, which bind to ESC via affinity and specificity, are also summarized. In addition, we review several markers that overlap with tumor stem cells (TSCs), which suggest that uncertainty still exists regarding the benefits of using these markers alone or in various combinations when identifying and isolating cells.

## 1. Introduction

Embryonic stem cells (ESCs) are pluripotent stem cells derived from the inner cell mass of the blastocyst, an early-stage embryo [1,2]. ESCs retain pluripotency and self-renewing ability due to both their inherent properties and the culture conditions in which they are propagated. The ability to differentiate into all cell lineages in living bodies while maintaining an undifferentiated state during *in vitro* culture makes ESCs prior to clinical transplantation. The key problem in clinical application of ESCs is to distinguish them from other cell types, especially tumor cells, to avoid potential risks. Unique gene expression patterns in cells result in the presence of molecule markers that can be used as the identification (ID) to distinguish a special cell type from others. There are many particular molecules that can affect the pluripotency and self-renewal of ESCs. Identification, characterization, and categorization of these molecules will provide useful tools for the identification and isolation of the ESCs and subsequent ESC studies.

In recent years, a wide range of cell surface markers and generic molecular markers have been reported to be indicative of undifferentiated ESCs, especially for human species. Proteins involved in several signal pathways are also known to have important functions in cell fate decision. Lectins and other similar peptides have been found to specifically bind to ESCs. Unfortunately, many ESC markers overlap with those of tumor stem cells, therefore it may become problematic when these markers are used for ESC identification and isolation. In addition, understanding the mechanisms that regulate the pluripotency of human ESCs (hESCs) remains a major challenge, as recent studies have shown that human and mouse ESCs differ in these mechanisms despite their similar embryonic origins [3]. Further knowledge of these markers is critically needed for the proper uses of ESCs and elucidation of the mechanisms governing the pluripotency and self-renewal of ESCs. 

**Figure 1:**
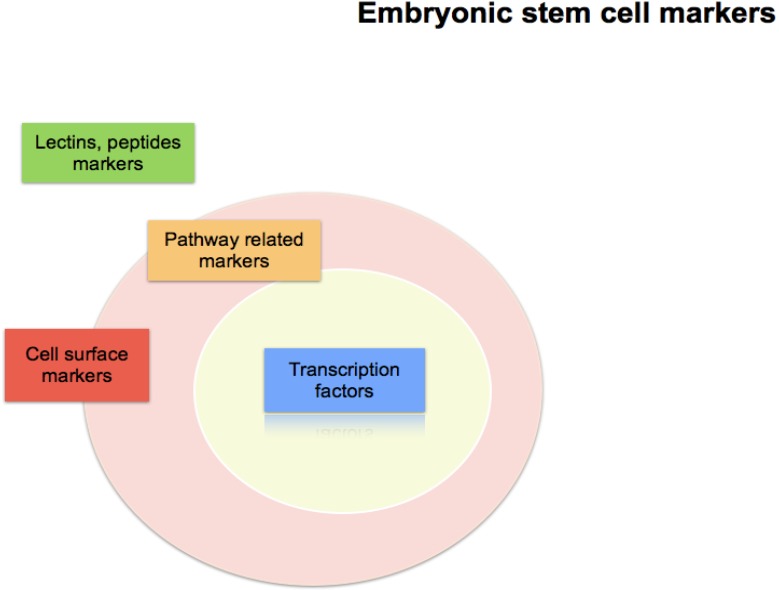
Categories of embryonic stem cell markers.

## 2. Cell Surface Markers

Coating the surface of cells are specialized proteins that can selectively bind or adhere to other signal molecules. Different types of membrane proteins differ in structure and in their affinity for signal molecules. Some of the proteins are uniquely present or secrete in specific cell types. Therefore, particular cell surface proteins can act as cell markers. Membrane proteins are the most important marker type in recognizing ESC without breaking the cell membrane (Table 1). However, most of the membrane markers are overlapping with tumor cell types (see section 7). 

**Table 1:** ESC surface markers.

SSEAs markers	Characteristics	Classification	References
SSEA-1 (CD15/Lewis x)	Murine embryos, mouse ES cells, mouse and human germ cells, embryonal carcinoma (EC) cells	Carbohydrate-associated molecules	[4,5,6,7,8,9,10,11]
SSEA-3	Primate ES cells, human embryonic germ cells, human ES cells, embryonal carcinoma (EC) cells	Carbohydrate-associated molecules	
SSEA-4	Primate ES cells, human embryonic germ cells, human ES cells, embryonal carcinoma (EC) cells	Carbohydrate-associated molecules	
**CD markers**			
CD324 (E-Cadherin)	Human ES cells, mouse ES cells, embryonal carcinoma (EC) cells	Surface marker (Binding to integrin alphaE/beta7, homotypic interactions mediate cell adhesion)	[12,13,14,15,16,17,18,19,20,21]
CD90 (Thy-1)	Human ES cells, mouse ES cells, hematopoietic stem cells, embryonal carcinoma (EC) cells	Surface marker (hematopoietic stem cell and neuron differentiation, T activation)	
CD117 (c-KIT, SCFR)	Human ES cells, mouse ES cells, hematopoietic stem progenitors, neural crest-derived melanocytes, primordial germ cells, embryonal carcinoma (EC) cells	Surface marker (Stem Cell Factor receptor)	
CD326	Human ES cells, mouse ES cells, embryonal carcinoma (EC) cells	Surface marker (function as growth factor receptor or adhesion molecule)	
CD9 (MRP1, TM4SF DRAP-27, p24)	Human ES cells, mouse ES cells	Surface marker (cell adhesion, migration, T co-stimulation)	
CD29 (β1 integrin)	Human ES cells, mouse ES cells	Surface marker	
CD24 (HAS)	Human ES cells, mouse ES cells	Surface marker (T co-stimulation, CD62P receptor)	
CD59 (Protectin)	Human ES cells, mouse ES cells	Surface marker (binds complement C8 and C9, blocks membrane attack complex assembly)	
CD133	Human ES cells, mouse ES cells, embryonal carcinoma (EC) cells, Hematopoietic stem cells	Surface marker	
CD31 (PECAM-1)	Human ES cells, mouse ES cells	Surface marker (CD38 receptor, signaling, platelet-endoth adhesion)	
CD49f (Integrin α6/CD29)	Human ES cells, mouse ES cells	Membrane receptors	[22,23,24,25,26,27,28,29,30,31]
**Markers**			
TRA-1-60	Human ES cells, teratocarcinoma, embryonic germ cells, embryonal carcinoma (EC) cells	Surface antigen	[8,32,33,34,35]
TRA-1-81	Human ES cells, teratocarcinoma, embryonic germ cells, embryonal carcinoma (EC) cells	Surface antigen	
Frizzled5	Human ES cells, mouse ES cells	Seven transmembrane-spanning G-protein-coupled receptor	[36,37,38,39,40]
Stem cell factor (SCF or c-Kit ligand)	ES cells, mouse ES cells, Hematopoietic stem cells, Mesenchymal stem cells, embryonal carcinoma (EC) cells	Cytokine, exist both as a transmembrane protein and a soluble protein	[41,42]
Cripto (TDGF-1)	Mouse ES cells, human ES cells, cardiomyocyte, embryonal carcinoma (EC) cells	Receptor for the TGF- β signaling pathway	[43,44]

### 2.1. Stage Specific Embryonic Antigens (SSEA)

SSEAs were originally identified by three monoclonal antibodies recognizing defined carbohydrate epitopes associated with the lacto- and globo-series glycolipids SSEA-1, SSEA-3, and SSEA-4 [5,45]. These carbohydrate-associated molecules are involved in controlling cell surface interactions during development. SSEA-1 (CD15/Lewis x) is expressed on the surface of murine embryos at the pre-implantation stage, as well as in mouse and human germ cells, and on the surface of teratocarcinoma stem cells, but it is absent in human ESC and human embryonic carcinoma cells [10,45]. SSEA-1 expression was also found in the oviduct epithelium, endometrium, and epididymis, as well as some areas of the brain and kidney tubules in adults [6]. SSEA-1 expression increases upon differentiation in human cells, decreases in differentiation in mouse. SSEA-3 and SSEA-4 are synthesized during oogenesis and are present in the membranes of oocytes, zygotes, and early cleavage-stage embryos [7,11]. They are expressed in undifferentiated primate ESC, human embryonic germ (EG) cells, human teratocarcinoma stem cells, and ESC [9]. SSEA4 expression is absent in murine ESC, but appears following differentiation [8,15]. 

### 2.2. Cluster of Differentiation (CD) Antigens

CD antigens are surface proteins that belong to several different classes, such as integrins, adhesion molecules, glycoproteins, and receptors. Different cell types have different CD antigens. Antibodies recognizing CD antigens are frequently used as an efficient tool in cell sorting and in identifying and characterizing various cell populations. Several CD antigens are associated with mouse and human ESC [19]. The CD antigens associated with pluripotent hES cells are CD9, CD24, and CD133 [12,13,14,16,17,18,14,16]. CD133 is also a hematopoietic stem cell marker [21]. In addition, hES cell express markers such as CD90 and CD117 [18,20,33]. However, CD133 and CD96 are also expressed in some tumor stem cells (see section 7).

#### 2.2.1. Integrins

Integrins are α/β heterodimeric cell surface receptors that mediate the attachment of a cell to its surrounding tissues. They play a pivotal role in cell adhesion, signaling, and migration, as well as in cell growth and survival [30]. Integrins work together with other proteins such as cadherins, immunoglobulin superfamily cell adhesion molecules, selectins, and syndecans, to mediate cell-cell and cell-matrix interaction and communication. They bind to cell surface and ECM components such as fibronectin, vitronectin, collagen, and laminin [26]. Not only integrins perform this outside-in signaling, but they also operate in an inside-out mode. The outside-in signaling via one integrin can promote the activation of another integrin via inside-out signaling [46]. Thus, they transduce information from the ECM to the cell as well as reveal the status of the cell to the outside, allowing rapid and flexible responses to changes in the environment. Multiple types of integrins exist on different cell surfaces, and they play an important role in constructing ESC niches [31]. The integrin family contains at least 18 α- and eight β-subunits that form 24 known integrins with distinct tissue distributions and overlapping ligand specificities [24]. The α5β1, αvβ5, α6β1, and α9β1 integrins play important roles in the maintenance of stemness in undifferentiated mouse ESC [27]. Integrin α6 (CD49f/CD29) is a 120-kDa protein with two splice variants, integrins α6A and α6B, which functions as a receptor for laminins and mediates cellular adhesion events on the basal membrane [22,25,48]. Integrin α6 (CD49f/CD29) plays an important role in hematopoietic stems and progenitor cells homing to the bone marrow [23,28] and human prostate carcinoma cells [29].

### 2.3. TRA-1-60 and TRA-1-81

TRA-1-60 and TRA-1-81 antigens on the human embryonal carcinoma (EC) cells [32] and human pluripotent stem cell surfaces are widely used as markers in identifying and isolating ESCs. They are also expressed in teratocarcinoma and EG cells [8,33,35]. TRA-1-60 antibody reacts with a neuraminidase-sensitive epitope of a proteoglycan, whereas TRA-1-81 reacts with a neuraminidase-insensitive epitope of the same molecule. Recently, this proteoglycan molecule has been proposed as a form of the protein podocalyxin [35]. Unfortunately, TRA-1-60 is also detected in the serum of patients with germ cell tumors [32,34]. 

### 2.4. Frizzled (Fzd)

Fzd belongs to the seven-transmembrane-spanning G-protein-coupled receptor (GPCR) superfamily [48]. Fzd has a large extracellular N-terminal region containing a cysteine-rich domain (CRD), which is involved in the binding to Wnt proteins [36,40]. Wnt signals are transduced through the FZD family receptors receptors [37]. The intracellular C-terminus of Fzd binds to the PDZ domain of Dvl proteins, a major signaling component downstream of Fzd. Wnt proteins bind to Fzd and the co-receptors LRP5 or LPR6, and activate the Wnt/β-catenin pathway by inhibiting the phosphorylation of β-catenin by GSK3-β. In addition to this canonical Wnt/β-catenin pathway, some Wnt proteins can also activate the Fzd/Ca^2+^ and Fzd/PCP (planar cell polarity) pathways. The mammalian Fzd subfamily has 10 members (Fzd1 to Fzd10) and may mediate signaling through different pathways. Some Fzds can also bind to other secreted proteins, such as Norrin and R-Spondin. Fzd 1-10 are expressed in mouse and human ESC [38,39]. 

### 2.5. Stem Cell Factor (SCF or c-Kit Ligand)

Stem Cell Factor (also known as SCF, kit-ligand, KL, or steel factor) is a cytokine that binds to the c-Kit receptor (CD117). SCF can exist both as a transmembrane protein and a soluble protein. Soluble SCF exists as a non-covalently associated homodimer is glycosylated, and has considerable secondary structure, including regions of alpha helices and beta sheets. Each SCF monomer contains two intra-chain disulfide bridges, and the N-terminal 141 residues of SCF have been identified as a functional core, SCF1-141, which include the dimer interface and portions that bind and activate the receptor Kit. SCF transduces signals by ligand mediated dimerization of its receptor, Kit, which is a type III receptor protein-tyrosine kinase related to the receptors for platelet-derived growth factor (PDGF), macrophage colony-stimulating factor, Flt-3 ligand and vascular endothelial growth factor (VEGF). Binding of SCF to Kit leads to receptor dimerization and activation of protein kinase activity [41]. Various fibroblast-type cells, and sites where hematopoiesis takes place, such as the fetal liver and bone marrow, all express SCF. This cytokine plays an important role in blood cells formation (hematopoiesis), spermatogenesis, and melanogenesis [44,49], it is reported that the survival of differentiating embryonic stem cells is dependent on the SCF-KIT pathway [42]. 

### 2.6. Cripto (TDGF-1)

The Cripto (also known as teratocarcinoma-derived growth factor-1, TDGF-1) gene encodes a novel human growth factor structurally related to epidermal growth factor. During development, Cripto acts as an obligate co-receptor for transforming growth factor β (TGF-β) ligands, including nodals, growth and differentiation factor 1 (GDF1), and GDF3. In addition to having essential functions during embryogenesis, as an oncogene, Cripto is highly expressed in tumors and promotes tumorigenesis via mechanisms including activation of mitogenic signaling pathways and antagonism of activin signalling [43,50,51].

## 3. Transcription Factors

Genes that function in the nucleus are always involved in important functions. Transcription factors are crucial for gene regulation. Some of these transcription factors are present in an inactive form under normal conditions, and only bind to their cognate recognition sequences when a specific signal transduction event happens. Unique genes appear and do functions in the nucleus means that the cell has responded to a certain condition. So, tracking these genes expression can be used as a marker for a specific cell situation. Transcription factors that expressed in ESC are listed in Table 2. 

**Table 2:** Transcription Factors.

CORE Nuclear transcription factors	Characteristics	Classification	References
Oct-3/4 (Pou5f1)	Mouse ES cells, human ES cells, embryonal carcinoma (EC) cells	POU family Transcription factors	[52,53]
Sox2	Mouse ES cells, human ES cells, embryonal carcinoma (EC) cells, neural stem (NS) cells	POU family binder Transcription factors	[54,55]
KLF4	Mouse ES cells, human ES cells, embryonal carcinoma (EC) cells	Zinc-finger Transcription factors	[56]
Nanog	Mouse ES cells, human ES cells, embryonal carcinoma (EC) cells	Transcription factors	[57,58,59]
**Markers**			
Rex1 (Zfp42)	Mouse ES cells, human ES cells, embryonal carcinoma (EC) cells	Zinc-finger Transcription factor	[60,61,62]
UTF1	Mouse human ES cells, germ line tissues in mouse and human, embryonal carcinoma (EC) cells	Transcriptional coactivator	[63,64]
ZFX	Murine ES cells, human ES cells, hematopoietic stem cells, embryonal carcinoma (EC) cells	X-linked zinc finger protein; Probable transcriptional activators	[65,66]
TBN	Mouse, human inner cell mass	New class of proteins with an important function in development	[67]
FoxD3	Murine ES cells, human ES cells, embryonal carcinoma (EC) cells	Forkhead Box family, transcriptional regulator	[68,69,70]
HMGA2	Mouse ES cells, human ES cells	Architectural transcription factors	[71,72,73,74]
NAC1	Mouse ES cells, human ES cells	The POZ/BTB domain family, nuclear factor	[75,76,77]
GCNF (NR6A1)	Mouse ES cells, human ES cells, embryonal carcinoma (EC) cells	Nuclear receptor gene superfamily, nuclear receptor	[78,79]
Stat3	Murine ES cells, Human ES cells, embryonal carcinoma (EC) cells	Transcription factor	[80,81]
LEF1, TCF3	Mouse ES cells, Human ES cells, embryonal carcinoma (EC) cells	(HMG) DNA binding protein family, Transcription factor	[82]
Sall4	Murine ES cells, Human ES cells, embryonal carcinoma (EC) cells	Zinc finger transcription factor	[83,84]
Fbxo15	Mouse ES cells, early embryos, and testis tissue, embryonal carcinoma (EC) cells	F-box protein family, target of Oct3/4	[85]
**ECAT genes**			
ECAT11 (FLJ10884/ L1TD1)	Human ES cells, embryonal carcinoma (EC) cells	Downstream target of Nanog	[86]
Ecat1	Mouse oocytes, embryonal carcinoma (EC) cells	KH domain containing RNA binding protein	[87]
ECAT9 (Gdf3)	Human ES cells, embryonal carcinoma (EC) cells	TGFβ superfamily, BMP inhibitor	[88]
**Dppa genes**		Oct4-related genes	
Dppa5 (ESG1)	Mouse ES cells, Human ES cells, embryonal carcinoma (EC) cells	K homology RNA-binding (KH) domain	[89,90]
Dppa4	Mouse ES cells, Human ES cells, embryonal carcinoma (EC) cells	Nuclear factor	[91]
Dppa2 (ECSA)	Mouse ES cells, Human ES cells, embryonal carcinoma (EC) cells	DNA-binding protein	[92,93]
Dppa3 (Stella)	Mouse ES cells, human ES cells, embryonal carcinoma (EC) cells, primordial germ cells, oocytes, preimplantation embryos	Maternal factor	[94,95]

### 3.1. CORE Nuclear Transcription Factors

In 2006, Yamanaka *et al.* [96] showed that that pluripotent stem cells can be obtained from mouse embryonic fibroblasts by combined expression of four factors, namely, Oct4, c-Myc, Sox2, and Klf4. Induced pluripotent stem (iPS) cells can be derived from a wide range of somatic cells via the over-expression of a set of specific genes. The four transgenes (Oct3/4, Sox2, c-Myc, and Klf4) are strongly silenced in Nanog iPS cells [transgenic mice containing the Nanog-GFP (green fluorescent protein)-IRES (internal ribosome entry site)-Puror (puromycin resistance gene cassette into the 59 untranslated region) reporter construct]. However, c-Myc is unnecessary in the generation of human iPS cells, and approximately 20% of the iPS cells’s offspring developed tumors attributable to the reactivation of the c-Myc transgene [97]. Thus, a modified protocol for the generation of iPS cells that does not require the Myc retrovirus has been developed, which generates significantly fewer non-iPS background cells. The iPS cells reprogrammed from fibroblasts to a pluripotent state by expression of Oct4, Sox2, and Klf4 in the absence of c-Myc are consistently of high quality [98,99]. It has been proposed that, in inducing pluripotency, the number of reprogramming factors used can be reduced when somatic cells express appropriate levels of endogenous complementing factors. For example, adult mouse neural stem cells (NSC) express endogenous Sox2 and c-Myc at higher levels compared with those of ESC, therefore Oct4, together with either Klf4 or c-Myc, is sufficient to generate iPS cells from NSC [100]. Recently, it has been shown that the expression of the transcription factor Oct4 is sufficient to generate pluripotent stem cells from adult mouse NSC, demonstrating that Oct4 is required and sufficient to directly reprogram NS cells to pluripotency [101]. Similar results have been obtained using human neural stem cells (NSCs) [102]. 

#### 3.1.1. Octamer-binding Protein 4 (Oct4)

Oct4 (Oct3/4 or POU5F1) is a member of the Oct family of POU transcription factors. It plays a key role in regulating stem cell pluripotency and differentiation [52,53]. The POU domain in these transcription factors is critical for their functions; regions outside the POU domain are not critical for DNA binding and exhibit little sequence conservation [103]. Oct4 ortholog genes share a high degree of genomic structural organization and sequence conservation and have been identified in different mammalian species, including humans, bovines, mouse, and rats [61]. Oct4 is restricted to pluripotent and germ line cells and its expression is maintained in the inner cell mass (ICM) of blastocysts [4,104]. A differential expression of Oct4 is also observed during embryogenesis, when the ICM differentiates into epiblasts (primitive ectoderm and embryonic ectoderm) and hypoblasts (primitive endoderm and embryonic endoderm) at 4.5 dpc. Oct4 expression is maintained in the epiblast; however, as hypoblast cells differentiate into the visceral and parietal endoderms, the Oct4 protein levels transiently increase and then decrease to undetectable. During gastrulation, Oct4 expression is progressively repressed in the epiblast at 7.5 dpc. In addition, ESC, EC cells, and EG cells are pluripotent cell lines derived from the ICM, epiblasts, and PGCs, respectively, and also express Oct4 as long as they remain undifferentiated. Oct4 influences the expression of several genes during early development, including Sox2, Fgf4, Rex1, hCG, and Utf1.

#### 3.1.2. Sry-related High-mobility Group (HMG) Box-containing (Sox) Family

Sox2 belongs to the Sox gene families, which are HMG box transcription factors that interact functionally with POU domain proteins [55]. The Sox family of genes is involved in maintaining pluripotency, similar to Oct3/4. However, it is associated with multipotent and unipotent stem cells, while Oct3/4 is exclusively expressed in pluripotent stem cells. Sox2 is one of the first genes used for induction of iPS cells by different groups [96,105]. The Sox2 gene bears at least two regulatory regions that specifically function in pluripotent embryonic cells. It is expressed in human and mouse pre-implantation embryos, mES, hES, mEC, and hEC cells in a similar manner as Oct4. In later development, Sox2 is again co-expressed with Oct4 in post-migratory primordial germ cells [54]. Oct4, Sox2, and osteopontin are co-expressed in the same cells of early mouse embryos. However, other genes in the Sox family are also involved in the induction process. Sox1 yields iPS cells with efficiency similar to that of Sox2, and the Sox3, Sox15, and Sox18 genes also generate iPS cells, although with a decreased efficiency.

#### 3.1.3. Krupple-like Factor (Klf) Family

The Klf family regulates numerous biological processes, including cell proliferation, differentiation, development, and apoptosis. All KLF family members are characterised by their three Cys2 His2 zinc fingers located at the C-terminus, separated by a highly conserved H/C link. DNA binding studies demonstrated that the KLFs have similar affinities for different GC-rich sites, and can compete with each other for the occupation of such sites. KLFs also share a high degree of homology between the specificity protein (Sp) family of zinc-finger transcription factors and bind similar in a large number of genes. Klf5 (also called intestine-enriched Krüppel-like factor or Bteb2) is the founding member of a completely new class of proteins with an important function in development. It has reported that Klf5 directly regulates the transcription of Oct3/4 and Nanog, which are involved in ESCs renewal and pluripotency maintenance [106]. Klf4 and Klf2 are functionally redundant in the self-renewal and pluripotency of ESCs. They also can regulate the expression of other ESC pluripotency related transcription factors as Nanog, Tcl1, Esrrb, Sall4, Tcf3, Mycn and Fbxo15. But a study showed that individual Klfs are not important for the self-renewal of ESCs [107], in ESCs, these Klfs were extensively colocalized to specific genomic regions, the three Klfs converge and work together to regulating common targets [56]. 

#### 3.1.4. Nanog

Nanog is a transcription factor and plays a crucial role in the maintenance of pluripotency and self-renewal in mouse and human ESCs [58,659]. Chambers *et al.* [57] established a central role for Nanog in the transcription factor hierarchy that defines ESC identity. Nanog mRNA is found in pluripotent ES and EG cells, as well as in both mouse and human EC cells. Its expression is downregulated early during ESC differentiation, which is consistent with an intimate association with pluripotent stem cell identity. The restricted expression of Nanog coincides with the transient potential for ESC generation, which arises in the ICM and is lost during implantation. Mitsui [59] showed that Nanog is required in the maintenance of pluripotency in mouse epiblasts and ESC. Nanog is capable of maintaining ESC self-renewal independent of LIF/Stat3. Nanog-deficient ICMs failed to generate epiblasts and only produced parietal endoderm-like cells. Nanog-deficient ESC lost pluripotency and differentiated into extraembryonic endoderm lineage. Thus, a central role for Nanog as a transcription factor that defines ESC identity has been reported [57]. Nanog mRNA is present in pluripotent mouse and human cell lines, and absent from differentiated cells. In preimplantation embryos, Nanog is restricted to founder cells from which ESC can be derived. Endogenous Nanog acts in parallel with the cytokine stimulation of Stat3 to induce ESC self-renewal. An elevated Nanog expression from transgene constructs is sufficient for the clonal expansion of ESC, bypassing Stat3 and maintaining Oct4 levels. Cytokine dependence, multilineage differentiation, and embryo colonization capacity are fully restored upon transgene excision.

### 3.2. Reduced Expression 1 (Rex1 or Zfp-42)

The Rex1 gene encodes for a zinc finger family transcription factor, which is highly expressed in mouse and human ESCs. Rex1 protein contains four nuclein acid zinc finger motifs and an acidic domain [61]. Rex1 was first identified as a gene expressed in F9 EC cells and is down-regulated after retinoic acid (RA) treatment to induce differentiation. It is similar to Yy1, an evolutionally conserved component of the polycomb-related complex [60]. Its expression is highly specific in mouse and human ESCs and is a famous marker of pluripotency in various stem cells, such as multipotent adult progenitor cells and amniotic fluid cells [61,62]. Its detailed function is unknown, but it is reportedly dispensable in both the maintenance of pluripotency in ESC and the development of embryos. Using a conventional gene targeting strategy, a study showed that Rex1 function is dispensable for both the maintenance of pluripotency in ESCs and the development of embryos [108]. Thus, Rex1 should be regarded as just a marker of pluripotency without a functional significance, similar to the activity of alkaline phosphatase. A number of studies have been conducted on Rex1 (Zfp42) function in ESC differentiation. Rex1 double knockout ESC lines have been generated and microarray analyses have been performed to compare the wild-type (wt) and Rex1-/- cells cultured with and without LIF to identify potential Rex1 targets. The results showed disruption of the Rex1 gene enhanced the expression of ectoderm, mesoderm, and endoderm markers as compared to wild-type (Wt) cells, suggest Rex1 acts to reduce retinoic acid induced differentiation in ES cells [109]. 

### 3.3. Undifferentiated Embryonic Cell Transcription Factor (UTF1)

UTF1 is a transcriptional co-activator that interacts with the metal-binding motif of activation transcription factor-2 (ATF-2). UTF1 is a strongly chromatin-associated protein involved in the initiation of ESC differentiation. UTF1 knockdown in ES and carcinoma cells resulted in a substantial delay or blockage in differentiation [97]. UTF1 is mainly expressed in pluripotent ESC, tightly associated with chromatin in mouse and human ESC, and may be involved in maintaining an epigenetic environment necessary for the pluripotent state [63,64]. UTF1 gene carries a regulatory element that selectively interacts with a Oct3/4 and Sox-2 complex [110]. Oct4 and Sox2 regulate the expression of UTF1 [111,112]. The co-expression of UTF1 with the reprogramming factors c-Myc, Oct4, Sox2, and KLF4, along with the siRNA knockdown of p53, increased the efficiency of induced pluripotent stem cell generation by 100-fold [113]. UTF1 is expressed in ECs, ESC, and in germ line tissues in mice and humans, but could not be detected in any of the other adult mouse tissues tested. In normal mouse embryos, UTF1 mRNA is present in the inner cell mass, the primitive ectoderm, and the extra-embryonic tissues [114]. In mouse blastocysts, UTF1 expression is restricted primarily in pluripotent cells (ICM cells), and its expression is rapidly down regulated upon differentiation [61]. 

### 3.4. X-linked Zinc Finger Protein (ZFX)

The ZFX gene is expressed from the inactive X chromosome and is structurally similar to its homologue on the Y chromosome, ZFY [66]. Transcripts of ZFX and ZFY encode for proteins composed of a highly acidic amino-terminal domain and a carboxy-terminal zinc-finger motif commonly associated with nucleic acid-binding. Both ZFY and ZFX are possible transcriptional activators and may function in sex determination. Multiple alternatively spliced transcript variants of ZFX for different isoforms have been identified and may be functionally distinct [115]. Conditional gene targeting studies in mice have suggested that ZFX is also required in the self-renewal of embryonic and hematopoietic stem cells [65]. ZFX may also be involved in the proliferation and expansion of B cells, and may contribute to lymphocyte homeostasis [116]. 

### 3.5. Taube Nuss (Tbn)

Tbn is the founding member of a completely new class of proteins with an important function in development. It is highly conserved between humans and mice. Tbn is restricted to the inner cell-mass (ICM) cells, and necessary for the survival of the ICM cells [67]. It could also be detected in hECSs [61]. ICM cells die of apoptosis in the absence of the Tbn protein. Tbn mutant mice exhibit disturbances in the balance between cell death and cell survival in the early embryo, so that the pluripotent ICM cells all die of apoptosis while the trophectoderm cells survive. 

### 3.6. Forkhead Box D3 (FoxD3)

FoxD3 is a member of the Forkhead box family and is characterized by a winged-helix DNA-binding structure. It plays an important role in embryonic development [70]. This transcriptional regulator is required for the maintenance of pluripotency in the pre-implantation and peri-implantation stages of mouse embryonic development [69] and is also required for trophoblast formation [117]. FoxD3 is required for the maintenance of the mammalian neural crest; FoxD3 (-/-) mouse embryos fail around the time of implantation, with a loss of neural crest-derived structures [118]. FoxD3 also forms a regulatory network with Oct4 and Nanog to maintain the pluripotency of ESCs [68].

### 3.7. HMGA2

HMGA2 is an architectural transcription factor. It does not have a direct transcriptional activation capacity, but instead regulates gene expression by changing the DNA conformation via binding to the AT-rich regions in the DNA and direct interaction with other transcription factors. HMGA2 is abundantly and ubiquitously expressed and plays a crucial role during embryonic development [71,72,73,74]. HMGA2 promotes stem cell self-renewal, and its reduced expression is associated with stem cell aging [74,119]. The expression levels of HMGA2 are very low in normal adult tissues, but its over-expression or rearrangement is associated with many types of cancer [120,121,122,123].

### 3.8. Nucleus Accumbens-1 (NAC1)

NAC1 is a nuclear factor that belongs to the Pox virus and zinc finger/bric-a-brac tramtrack broad complex (POZ/BTB) domain family. Also known as BTBD14B, it was originally identified in a unique neuronal forebrain structure responsible for reward motivation and addictive behaviors [75,77]. NAC1 recruits HDAC3 and HDAC4 to transcriptionally repress gene expressions in neuronal cells and specifically co-represses other POZ/BTB proteins in the central nervous system [124,125]. NAC1 is upregulated in several tumor types, including breast, renal cell, and hepatocellular carcinoma, as well as high-grade ovarian serous carcinoma, where it has long been suspected as a chemoresistance gene [126,127]. The chemoresistance mechanism reportedly occurs through the NAC1 negative regulation of the GADD45 pathway [128]. NAC1 has also been described as part of the extended transcriptional network in pluripotent cells that involves Oct4, Sox2, Nanog, Sall1, KLF4, and Sall4 [76].

### 3.9. Germ Cell Nuclear Factor (GCNF)

GCNF, also known as the nuclear receptor subfamily 6 group A member (NR6A1), is an orphan member of the nuclear receptor gene superfamily [78]. It is expressed in the nervous system during development and during specific stages in the maturing germ cells of the adult ovary and testis in adults. In addition, it may also be involved in gametogenesis, neurogenesis, and normal embryonic development during gastrulation [78,79]. The inactivation of GCNF in mice results in an abnormal posterior development, impaired midbrain development, insufficient closure of the neural tube, and eventual embryonic death [129,130]. GCNF has been shown as a repressor of Oct4 and the protamine genes [131,132] and plays a critical role in the control of gene expression during embryogenesis and spermatogenesis [79,133].

### 3.10. Stat3

The Stat3 transcription factor is an important signaling molecule for many cytokines and growth factor receptors [81] and is necessary for murine fetal development [134]. The main extracellular signal that sustains mouse ESCs self-renewal and pluripotency is the activation of leukemia inhibitory factor (LIF) pathway, and by binding LIF to the LIF receptor, signals the activation of the Stat3 that translocates into the nucleus and activates a variety of downstream genes, including Sall4, Myc, and KLF4 [135]. Stat3 suppression results in ESC differentiation [136], whereas constitutive activation of Stat3 is sufficient to maintain ESCs in the undifferentiated state, even in the absence of LIF [137]. Stat3 is constitutively activated in a number of human tumors [138,139] and possesses oncogenic potential [141] and anti-apoptotic activities [138]. It is activated by phosphorylation at Tyr705, which induces dimerization, nuclear translocation, and DNA binding [141,142]. Transcriptional activation seems regulated by phosphorylation at Ser727 through the MAPK or mTOR pathways [143,144]. A Stat3 isoform expression suggests a biological function, given that the relative expression levels of Stat3α (86 kDa) and Stat3 β (79 kDa) depend on the cell type, ligand exposure or cell maturation stage [145]. 

### 3.11. LEF1 and TCF

LEF1 and TCF are members of the HMG DNA-binding protein family of transcription factors, which consists of the lymphoid enhancer factor 1 (LEF1), T-cell factor 1 (TCF1), TCF3, and TCF4 [146]. LEF1 and TCF1 were originally identified as important factors regulating early lymphoid development [82,147] and act downstream in Wnt signaling. LEF1 and TCF bind to Wnt response elements to provide docking sites for β-catenin, which translocates to the nucleus to promote the transcription of target genes upon activation of Wnt signaling. LEF1 and TCF proteins are dynamically expressed during the development and aberrant activation of the Wnt signaling pathway, which is involved in many types of cancers, including colon cancer [148]. TCF3 (also known as TCF7L1) plays an important role in integrating Wnt signaling with the regulation of stem cell differentiation [149,150].

### 3.12. SALL Family

The SALL gene family (also called Hsal), which has important roles in regulating the developmental processes of many organisms, comprises SALL1, SALL2, SALL3, and SALL4 and was originally cloned from a DNA sequence homologous to the Drosophila gene sal [83,151]. SALL4 is an important regulator of Oct4 and is required for the pluripotency of ESC [84]. Early embryonic cell-fate decisions must ensure the correct formation of the epiblast *in vivo*. Sall4 downregulation in mouse ESCs results in the respecification of the ESCs to the trophoblast lineage when the cells are grown in feeder-free conditions. Sall4 is reportedly essential for stabilization, but not for ESC pluripotency [152]. SALL4 and Oct4 work antagonistically to balance the expression of other SALL gene family members. Other SALL gene members, especially Sall1 and Sall3, are expressed in both murine and human ESC. The deletions of these two genes in mice lead to perinatal death due to developmental defects [153,154].

### 3.13. F-box 15 (FBXO15)

FBXO15 is a member of the F-box protein family, which is characterized by an approximately 40-amino acid F-box motif. FBXO15 is a novel target of Oct3/4, but is dispensable in ESC self-renewal, development, and fertility [85]. FBXO15 is predominantly expressed in undiffereniated mouse ESC. The inactivation of Oct3/4 in ESC leads to the rapid disappearance of FBXO15 expression. The expression profile of FBXO15 is nearly identical to that of Oct3/4, and is restricted in ESC, early embryos, and testicular tissue.

### 3.14. ESC Associated Transcript (ECAT) Genes

ECAT genes are a set of genes that play an important role in stem cell biology. ECAT1 encodes for a K homology RNA-binding (KH) domain containing an RNA-binding protein specifically expressed in mouse oocytes [87]. ECAT4 was identified as Nanog, a master regulator for the maintenance of mESC and hESC [59]. ECAT5 was identified as an ESC-expressed Ras (ERas), a Ras-like oncogene important in regulating mESC proliferation [155]. ECAT9 was identified as growth and differentiation factor 3 (GDF3), an important factor that helps maintain mESC pluripotency by inhibiting bone morphogenetic protein (BMP) signaling [88]. ECAT11, also named FLJ10884 or L1TD1, is abundantly expressed in undifferentiated hESC. ECAT11 has demonstrated that L1TD1 is a downstream target of Nanog and represents a useful marker in identifying undifferentiated human ESC [86]. 

### 3.15. Developmental Pluripotency-associated (DPPA) Genes

DPPA molecules are a group of five proteins related by names only, described as a set of Oct4-related genes. They serve as markers for early embryonic and germline pluripotent cells. DPPA5, also named ESG1, is a KH domain containing protein expressed in EG cells and ESC. It is also a potential marker for ESCs [89,90]. DPPA3, also called Stella, is expressed in primordial germ cells, oocytes, preimplantation embryos, and pluripotent cells [92,93]. DPPA3 is a marker of pluripotency and plays a role in transcriptional repression, cell division, and maintenance of cell pluripotentiality in mice and humans. Related intron-less loci are expressed in germ cell tumors [94,95]. DPPA4 is reported as a nuclear factor associated with active chromatin and that it regulates differentiation of ESCs into a primitive ectoderm lineage [91].

## 4. Signal Pathway-related Intracellular Markers

Usually, the best-characterized markers exist on the cell surface. An external signal that could not smoothly penetrate through the cellular membrane must interact with cell surface molecules to transmit signals. Once a specific intracellular signal pathway is activated, it will trigger a series of molecular events that leads the regulations of specific gene expressions in response to this signal. Several intracellular signal pathways play important roles in maintaining ESC self-renewal and pluripotency; therefore, many signal pathway-related proteins, which are critical for these roles, therefore, can be regarded as ESC markers. Here we discuss several signal pathway related proteins that are known to have important functions in ESC fate. 

LIF-STAT3, BMP-SMAD, TGF- β /Activin/Nodal, IGF-IR, FGFR and Wnt-β-catenin may be considered as the core signal pathways in regulating ESC self-renewal and pluripotency [156,157,158,159]. LIF-STAT3 and BMP-SMAD has been shown to be critical for mouse ES cell self-renewal [160,161], but LIF-STAT3 is not active in undifferentiated hESC [162,163,164]. Although BMP signal pathways play a significant role in both mouse and human ESCs [165,166,167,168], the upstream effectors and effects of signaling frequently differ. For instance, BMP4 has been shown to block the differentiation of mESC and maintain pluripotency [157], but it induces the trophectoderm differentiation of hESC [169,170]. BMP signaling pathways transduce signals via the SMAD protein. The activated SMAD protein regulates downstream gene expressions by combining with other DNA-binding proteins in the nucleus, which are expressed in ESCs with high levels [166,171,172,173,174]. Thus, the SMAD proteins related to these pathways, such as SMAD1/5/8, may serve as a marker gene for ESC.

The Wnt and TGF- β /Activin/Nodal pathway are also critical to self-renewal in both mouse and human ES cells. As a regulator, Wnt/β-catenin may be involved in many aspects of cell growth and embryonic development [175,176]. It has been demonstrated that Wnt/β-catenin expresses in high levels in ESCs and can regulate their pluripotency [177,178]. Therefore it can be considered as a marker. Ludovic Vallier *et al*. [179] have shown that TGF-β family members are involved in the cell fate decision of hESC. They also reported that activin/nodal signaling via Smad2/3 and Smad4 activation is necessary to maintain the pluripotent status of hESC, and Activin/Nodal has been shown to increase the transcription of Oct4 and nanog [180]. Therefore, Smad2/3 and Smad4 may also be considered as a marker in hESC. The potential markers among these pathways are shown in Table 3.

**Table 3:** Signal pathway-related intracellular markers.

Markers	Characteristics	Classification	References
SMAD1/5/8	Mouse ES cells, embryonal carcinoma (EC) cells	Smad proteins ((R-Smad), BMP signalling pathway	[171,173]
SMAD4	Mouse ES cells, human ES cells, embryonal carcinoma (EC) cells, early embryos, and testis tissue	Smad proteins (Co-SMAD), TGF- β /Activin/Nodal signalling pathway, BMP signalling pathway	
SMAD2/3	Human ES cells, embryonal carcinoma (EC) cells	Smad proteins ((R-Smad), TGF- β /Activin/Nodal signaling pathway	
β-catenin	Mouse ES cells, human ES cells, embryonal carcinoma (EC) cells	Transcription activators, Wnt/β-catenin signaling pathway	[177,178]

## 5. Enzymatic Markers

Both mouse and human ESCs express high levels of *alkaline phosphatase* and *telomerase*. ESC have elevated levels of alkaline phosphatase on their cell membrane, in humans, TRA-2-49 and TRA-2-54 antibodies can detect the alkaline phosphatase. In murine cells, they are typically visualized by an enzymatic-based reaction [61]. Therefore alkaline phosphatase staining is used to detect these cells and to test pluripotency. Some of these markers are listed on NIH stem cell resource web site (http://stemcells.nih.gov/info/scireport/appendixe.asp#eii).

## 6. Other Markers

Recently, researchers have been using small molecules such as lectins or short peptides that specifically bind to the receptors on the surface of ESC. Labeled with quantum dots (QD) or fluorescence dyes, these small molecules, binding specifically to ESC surface proteins, can act as markers to label, identify, and isolate ESC.

### 6.1. Lectins

Lectins are carbohydrate-binding proteins that recognize diverse sugar structures and have been extensively used to identify and characterize cell surface glycosylation patterns [181]. Studies of lectins have led to the delineation of embryologic developmental stages in some species, and lectins have been used to investigate and identify cell types based on the presentation of specific cell surface carbohydrates [182,183,184,185]. Many developmentally regulated glycans identified as lectin receptors in mouse ESC are displayed on cell surfaces at the preimplantation and implantation stages of development [186]. Lectins have been used as a marker to identify the mouse ESC-derived retinal progenitor cells for transplantation therapy [186] and for probing differentiated human ESCs [187,188]. Lectin can also be used as a marker to define the stages of mouse embryogenesis. Lectins can also provide a source of unique markers for the characterization of subpopulations that exist in colonies of adherent hESC. 

### 6.2. Peptides Specific for ES Cells

Receptor-ligand interactions are the basis for most cellular biological processes. Finding ligands that bind to a specific cell target is fundamental to the development of pharmaceutical drugs, biomaterials, and diagnostic tools [189]. Phage display technology [190] is one of the most efficient methods to identify novel biomarkers. The principle of the technology is based on fusing nucleotide sequences of random polypeptides to that of a phage coat protein that enables display of the chimeric proteins on the phage surface. By selection with the target of interest, a phage pool that has increasing specific binding ability to the target can be obtained efficiently. Ligands found from a phage display screen can bind to specific sites of target cells and can be used as markers to recognize and isolate cells. A series of small peptides specific to Rhesus Monkey Embryonic Stem Cells (R-ESCs) and mouse ESCs has been reported by our group [191,192], conjugated with quantum dots that the peptide (APWHLSSQYSRT) could target ESCs. And also, peptides specific to human ESCs and human embryonal carcinoma cells (ECs) have been reported [189]. When ESCs were cultured on self-assembled monolayers (SAMs) presenting the sequence TVKHRPDALHPQ or LTTAPKLPKVTR in a chemically defined medium (mTeSR), they expressed markers of pluripotency at levels similar to those of cells cultured on Matrigel [189]. The small peptide sequences related to ESCs are shown in Table 4.

**Table 4:** ESC specific peptide markers.

Mouse ES cells [191]	Macaca ES cells RS366.4 [192]	Human embryonal carcinoma (EC) cells [189]	Human embryonic stem cell line H9 [189]
GTYNLPNPPPPL	APWHLSSQYSRT	LTTAPKLPKVTR	APWHLSSQYSRT
KHMHWHPPALNT	GYPHPWTLWHLN	TVKHRPDALHPQ	DLNYFTLSSKRE
SAHGTSTGVPWP	LDVRPWYVTPLP	QLGTQPNSRTYA	HGEVPRFHAVHL
VPTATLMGASAR	WAPEKDYMQLMK	SRYITTMNTEQV	NRQSNWPIHKTI
WAETWPLAQRPP	TPLINMNALTVT	VTSRTIIPQGSA	QLSEECSYLISRP
LSTHTTESRSMV		TVKHRPDALHPQ	QLTKNVPTYKSS
SGHQLLLNKMPN		FAKSPDVSLNPS	SNPQPYTILPPV
THAAHMGYPSWW			SPLITSTLIPQR
LLADTTHHRPWT			SPNQPYTILPPV
			TALATSSTYDPH
			TPLTLRTQTLTQ
			TTKQPHFHQKTL
			TTLVSTGQRTHP

## 7. Markers Overlapping with Tumor Stem Cells

The ability of adult stem cells to live long and self-renew, as well as their multilineage differentiation, makes these cells unique and important in normal physiological and pathological conditions [193]. If a stem cell differentiation potential becomes impaired and proliferative capacity becomes uncontrolled, these mutated, self-renewing stem cells may become potentially tumorigenic and cause cancer [194]. Cancer stem cells (CSC) or tumor stem cells (TSC) are considered important components in carcinogenesis. CSCs have been isolated from many organs, such as the breast, brain, blood (leukemia), skin (melanoma), head and neck, thyroid, cervix, and lungs [195,196,197]. In recent studies, numbers of CSC markers were used to identify tumor cells from normal tissues [198,199,200]. However, ESC and CSC share many common marker genes, therefore causing the potential risk for ESC transplant. We summarized the recently identified CSC markers, shown in Table 5, and compared them with ESC makers.

SSEA-1 (CD15/Lewis x), a cell surface marker for neural stem cells (NSCs), functions in brain tumor stem cells (BTSC), including self-renewal, multidifferentiation, and the ability to recapitulate the phenocopy of primary tumors [201]. SSEA3 has been demonstrated expressed in breast cancer cells, including BCSCs, as well as in various normal tissues such as kidney [202]. Furthermore, the expression of SSEA3 was mostly restricted to the cytoplasm or apical surface of epithelial cells. Similar to that of Globo H. gene, the levels of SSEA3 and SSEA4 expression in breast cancer are much higher than that in benign breast lesions [202]. TRA-1-60 has been related to Germ cell tumor [34]. CD133 is expressed in many tumors and plays a significant role in the identification of CSCs [195,203]. A recent study revealed that CD133 is distributed in pancreatic exocrine cancer [204]. In the colon, CD133 expression is a marker for colon cancer with high prognostic impact, although it seems to have no obvious functional role as a driving force in this malignancy [205]. CD96 is a promising candidate as a leukemic stem cell (LSC)-specific antigen and is expressed on the majority of CD34^+^CD38^−^ Acute myeloid leukemia (AML) cells, whereas only a few cells in the normal Hematopoietic stem cell (HSC)-enriched population express CD96 weakly [206]. The CD90+ cells from hepatocellular carcinoma (HCC) cell lines display tumorigenic capacity. CD45-CD90+ cells can generate tumor nodules in immunodeficient mice [207]. CD326 (EpCAM) is a transmembrane glycoprotein mediating epithelial-specific intercellular cell-adhesion, and it is also involved in cell signaling, migration, proliferation and differentiation. CD326 has been found in human colon carcinoma, and most adenocarcinomas, and it is involved in tumor metastases, malignant effusions, and cancer stem cells [208]. CD9 was expressed preferentially in Small cell lung cancer (SCLC) and metastasized tumors. It is related to the cell adhesion-mediated drug resistance mechanism, highlighting it as an attractive therapeutic target to improve therapeutic outcomes in SCLC [209]. Recent research suggests that CD9 can be potential prognostic markers of gastric GIST and may serve as novel therapeutic targets of gastrointestinal stromal tumor (GIST) [210]. Knockdown of CD9 remarkably reduces the leukemogenic potential, suggesting that CD9 is related to several signaling pathways and epigenetic modification for regulating the CSC properties of B-acute lymphoblastic leukemia [211]. CD55 play an important role in tumorigenesis of breast cancer, and presence of small population of cells with strong CD55 expression would be sufficient to predict poor prognosis in patients [212]. In non-small cell lung cancer (NSCLC) cells, CD55 and CD59 were closely correlated with histological types, prognosis and preoperational adjutant chemotherapy of the disease. The expression of CD55 and CD59 are related to herceptin-induced complement-dependent cytotoxicity (CDC), and also promote suppressed anti-tumor effectiveness of herceptin, suggesting that CD55 and CD59 may be useful markers for predicting the clinical response to Herceptin therapy [213]. Furthermore, CD59 gene is highly expressed in many cancers such as ovarian and prostate carcinoma [214]. Recent researches about Hepatitis B X-interacting protein (HBXIP) function on the breast cancer cells has been shown that CD55, CD59, and CD46 are up-regulated through p-ERK1/2/NF-jB signaling to protect breast cancer from complement-dependent cytotoxicity [215]. GDF3-driven CD24 acts as a receptor for endogenous innate immune ligands that modulate cell proliferation; CD24 is an effective determinant of tumorigenesis in malignant cell transformation [216]. 

CD Markers cannot only indicate the growth of tumor stem cells, but also their invasion and metastasis. CD44^+^/CD24^−/low^ cells have been identified in breast carcinoma, in which they exclusively retain tumorigenic activity and display stem cell-like properties. They can induce breast cancer cells to propagate *in vitro* [217]. The hyaluronan receptor CD44 plays an important role in facilitating the invasion and metastasis of a variety of tumors. CD44 signaling is involved in underpinning breast tumor invasion, thereby promoting breast tumor invasion and metastasis to the liver [218]. In prostate cancer cells (PCas), CD44+ PCa cells are more proliferative, clonogenic, tumorigenic, and metastatic than the isogenic CD44^−^ PCa cells. The CD44+ PCa cell population is enriched in tumorigenic and metastatic progenitor cells [219].

Among the transcription factors, Oct-4 is highly expressed in bladder cancer [220]; Sox2 is found in breast tumours and activated in breast cancer stem cells [221]; Nanog has been shown to be related to brain and other kinds of cancer stem cells [194]; and KLF4 is required for maintenance of breast cancer stem cells and for cell migration and invasion [222]. Rex1 (Zfp42) is expressed in most cultured normal human epithelial cells, and human Rex1 mRNA expression is significantly reduced or lost in most human cancer cell lines [223]. Furthermore, Rex1 mRNA and protein levels are significantly decreased in most renal cell carcinoma specimens [224]. The discovery of Rex1 expression in meiotic cells from both testes and ovary indicate its role in meiosis. UTF-1 plays a possible role in spermatogonial self-renewal. UFT-1 and Rex1 are expressed in Testicular germ cell tumors (TGCT), and the high abundance of UTF-1 in spermatocytic seminomas is consistent with the hypothesis that this tumour type originates from spermatogonia [225]. A recent study shows that ZFX is differentially expressed in gastric cancer [226]. HMGA2 gene is found to expressed in pancreatic adenocarcinoma [227] and up-regulated in bladder cancer at both the transcriptional and translational levels compared with normal bladder tissue [228]. The expression of Embryo-cancer sequence A (ECSA), also named developmental pluripotency associated-2 (DPPA2), is limited to normal testis, placenta, bone marrow, thymus, and kidney, but it is expressed in a variety of tumors, most notably in 30% of non-small cell lung cancers (NSCLC) [229]. The LEF1 gene is frequently mutated in its NH2 terminus in human sebaceous tumors, and these mutations play a dual role in skin cancer, specifying tumor type by inhibiting Wnt signaling and acting as a tumor promoter by preventing induction of p53 [230]. LEF1 inactivation is an important step in the pathogenesis of T-cell acute lymphoblastic leukemia. LEF1 is highly expressed in androgen-independent prostate cancer [231]. 

Several other markers are also expressed in many tumor tissues, such as Cripto, NCA1, and SATA3. There are some differences between CSC and ESC, such as Nanog gene expressions, it was showed the most differentially expression in many glioblastoma stem cells. The expression levels of Nanog in normal neural stem cell (NSC) and in differentiated tumor cells are negligible [194]. Thus, it is a reliable way of differentiating a tumor from non-TS cells within glioblastoma multiforme (GBM) tumors [194].

The reported tumor stem cell markers also included Aldehyde dehydrogenase (ALDH1) [232], Musashi-1 [233], LgR5 [234], the prostate stem cell antigen (PSCA) [235], Doublecortin and CaM kinase-like-1 (DCAMKL-1) [236], and acute myeloid leukemia (AML) stem cell-surface marker TIM3 [237]. CD44+/CD24- cells represent a population that correlates with human breast CSCs [238]. The stromal cell-derived factor-1 (SDF-1) and its receptor, CXCR4, have been reported to be related to stem cell homing and cancer cell metastasis. It has been shown that CXCR4/SDF-1 may sustain tumor chemotaxis in CSC. The expression of CXCR4 was also detected in CD133+ cancer cells [239]. 

**Table 5:** Tumor stem cell markers.

Overlapping with ESCs	Characteristics	Classification	References
SSEA-1 (CD15/ Lewis x)	Brain tumor stem cells	Surface marker	[240]
SSEA-3	Breast cancer stem cells, breast Cancer	Surface marker	[202]
SSEA-4	Breast Cancer, epithelial ovarian carcinoma	Surface marker	[241]
TRA-1-60	Germ cell tumor	Surface marker	[32,34]
CD133	Pancreatic exocrine cancer; colon cancer; glioma;	Surface marker	[204,242,243,244,245]
CD29	Mouse mammary cells	Surface marker	[246]
CD24	Breast tumor, tumor invasion, prostate cancer	Surface marker	[217,218,247]
CD90	Hepatocellular carcinoma cell lines	Surface marker	[248]
CD9 (DRAP-27, MRP-1, p24)	Small cell lung cancer	Tetraspanin superfamily	[209,210,211]
CD59	Lung cancer, breast cancer	Membrane attack complex inhibition factor	[213,214,215]
CD326 (EpCAM)	Hepatocellular carcinoma	Wnt-B-catenin signaling target gene	[249]

Nanog	Brain and other kinds of cancer stem cells	Transcription factors	[194]
SOX2	Breast cancer stem cells, breast tumor	Transcription factors	[221]
OCT4	Bladder cancer, Lung Cancer stem cells	Transcription factors	[220,250]
KLF4	Breast cancer stem cells	Transcription factors	[222,251]
Rex1 (Zfp42)	Prostate Cancer, Renal cell carcinoma	Zinc-finger protein-42	[223,224]
UTF-1	Testicular germ cell tumours	Undifferentiated embryonic cell transcription factor 1	[225]
ZFX	Gastric cancer	Zinc-finger protein family	[226]
LEF1	Prostate cancer, lymphoblastic leukemia	Lymphoid enhancer-binding factor	[230,231,252]
SALL4	Breast cancer, leukemogenesis, testicular germ cell tumors	Transcription factor	[253,254,255]
ECAT9 (Gdf3)	Melanoma, Breast Carcinoma, Seminoma, germ cell tumors	TGFβ superfamily, BMP inhibitor	[216,256,257]
DPPA2 (ECSA)	Lung Cancer	DNA-binding protein	[229]
HMGA2	Pancreatic adenocarcinoma, bladder cancer	Architectural transcription factors	[227,228]
NAC1	Breast, renal cell, and hepatocellular carcinoma	Nuclear factor	[126,127]

Cripto	Broad range of tumors	Extra-cellular plasma membrane growth factor	[14,17]
Stat3	A number of human tumor	Transcription factor	[138,139,258]
Tumor stem cell markers			
ALDH1	Lung tumor	Surface marker	[232]
Musashi-1	Endometrium tumor stem cells	Neural stem cell regulatory protein	[233]
LgR5	Esophageal adenocarcinomas	G-protein coupled receptor	[234]
DCAMKL-1	Intestinal neoplasia; adenoma stem cells	Related to β-catenin	[236]
TIM3	Myeloid leukemia (AML) stem cell	Surface marker	[237]
Brca1	Mammary tumor	Human caretaker gene	[238]
SDF-1, CXCR4	Homing of stem cells and metastasis of cancer cells	Cell factor	[239]
PSCA	Prostate cancer	Cell surface antigen	[235]
CD96	leukemic stem cells	Surface marker	[206]
CD44	Breast tumor, tumor invasion, prostate cancer	Surface marker	[217,218,247]
CD45	Hepatocellular carcinoma cell lines	Surface marker	[248]

## 8. Conclusions and Future Prospects

**Figure 2:**
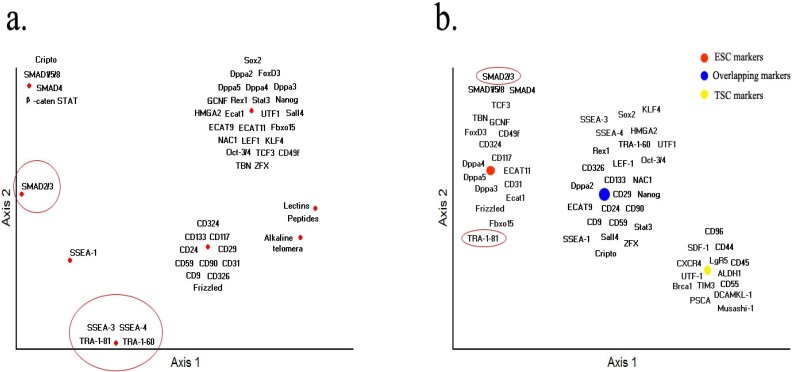
Non-metric multidimensional scaling (NMS) ordination. (**a**) The expression patterns of markers in ESC. To identify the expression profiles of markers in embryonic stem cells, similarity matrix was computed on the basis of presence/absence of such parameters as mouse embryonic stem cells, human stem cells, nuclear, cytoplasm, surface, enzymatic and out of cell. (**b**) The expression patterns of markers between ESC and TSC. Similarity matrix was computed merely based on the presence/absence. NMS was performed using Medium Autopilot and others as defaulted. Distance matrix was calculated based on Euclidean.

On all the markers described above, we performed non-metric multidimensional scaling (NMS) ordination analysis according to the expression profiles of markers in embryonic stem cells, based on the basis of presence/absence of such parameters as mouse embryonic stem cells, human stem cells, nuclear, cytoplasm, surface, enzymatic and out of cell. The results suggest five markers are critical only for human ESCs, including surface marker genes SSEA-3, SSEA-4, TRA-1-60, TRA-1-81 and pathway related gene SMAD 2/3, as shown in Figure 2a (circled in red). SSEA-1, STAT3 and SMAD1/5/8 are only important for mouse ESC. Most of the CD markers reported have also been related to tumor cell types except CD324, CD117, CD31 and CD49f, but future studies are needed to confirm that these CD genes are truly not related to tumors. Because TSCs and ESCs utilize the similar regulatory mechanicals, most of the key transcriptional factors and pathway related genes functions similarly in both TSCs and ESCs. The result also shown that TRA-1-81 and SMAD 2/3 may act as good candidates for human marker-based sorting (Figure 2b, circled in red). Interestingly, reduction in SMAD 2/3 signaling enhances tumorigenesis breast cancer cell lines [259]. Currently it is unknown whether lectins and other small peptides are linked with tumor cells. 

Understanding the mechanisms that regulate the pluripotency of ESC remains a major challenge, especially because recent studies have shown that human and mouse ESC differ in these mechanisms despite their similar embryonic origins. Although uncertainty still exists regarding the benefits of using these markers alone or in various combinations for cell identification and isolation, new scientific and technological development will lead to new breakthrough in stem cell marker studies, and more new markers will be discovered and effectively utilized.
